# Bimetallic NiCo_2_S_4_ Nanoneedles Anchored on Mesocarbon Microbeads as Advanced Electrodes for Asymmetric Supercapacitors

**DOI:** 10.1007/s40820-019-0265-1

**Published:** 2019-04-23

**Authors:** Yu Zhang, Yihe Zhang, Yuanxing Zhang, Haochen Si, Li Sun

**Affiliations:** Beijing Key Laboratory of Materials Utilization of Nonmetallic Minerals and Solid Wastes, National Laboratory of Mineral Materials, School of Materials Science and Technology, China University of Geosciences, Beijing, 100083 People’s Republic of China

**Keywords:** Bimetallic sulfides, NiCo_2_S_4_, Nanoneedles, Mesocarbon microbeads, Asymmetric supercapacitor

## Abstract

Bimetallic Ni–Co sulfides are outstanding pseudocapacitive materials with high electrochemical activity and excellent energy storage performance as electrodes for high-performance supercapacitors. In this study, a novel urchin-like NiCo_2_S_4_@mesocarbon microbead (NCS@MCMB) composite with a core–shell structure was prepared by a facile two-step hydrothermal method. The highly conductive MCMBs offered abundant adsorption sites for the growth of NCS nanoneedles, which allowed each nanoneedle to fully unfold without aggregation, resulting in improved NCS utilization and efficient electron/ion transfer in the electrolyte. When applied as an electrode material for supercapacitors, the composite exhibited a maximum specific capacitance of 936 F g^−1^ at 1 A g^−1^ and a capacitance retention of 94% after 3000 cycles at 5 A g^−1^, because of the synergistic effect of MCMB and NCS. Moreover, we fabricated an asymmetric supercapacitor based on the NCS@MCMB composite, which exhibited enlarged voltage windows and could power a light-emitting diode device for several minutes, further demonstrating the exceptional electrochemical performance of the NCS@MCMB composite.

**Electronic supplementary material:**

The online version of this article (10.1007/s40820-019-0265-1) contains supplementary material, which is available to authorized users.

## Highlights

- A facile method was used to anchor pseudocapacitive bimetallic NiCo_2_S_4_ (NCS) nanoneedles on mesocarbon microbeads (MCMBs), forming a novel urchin-like structure.
- The NCS@MCMB electrode and an assembled asymmetric supercapacitor displayed good electrochemical performance.

**Electronic supplementary material:**

The online version of this article (10.1007/s40820-019-0265-1) contains supplementary material, which is available to authorized users.

## Graphical abstract

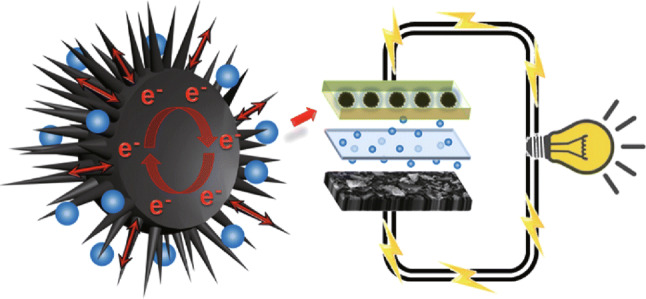

## Introduction

In the last few decades, the growing demand for high-performance mobile devices and renewable energy has promoted the continuous development of energy storage devices [1–5]. Supercapacitors have shown potential applications in energy storage, owing to their high power density, short charge–discharge time, long cycle life, and environmental friendliness [6–14]. The electrochemical performance of supercapacitors is strongly dependent on the electrode materials, which is also the key element for overcoming the limited energy density issues affecting supercapacitors. From the viewpoint of electrochemical performance, an ideal electrode material is expected to provide high specific capacitance and outstanding electrochemical stability. Despite their high electrical conductivity and long cycle life, traditional carbon materials exhibit capacitances based on the electrochemical double-layer capacitor (EDLC) mechanism; therefore, they cannot provide the large energy densities required by advanced energy devices [15–17]. In comparison, the specific capacitance of pseudocapacitive materials such as conducting polymer and transition metal compounds generally originates from reversible redox reactions; therefore, these materials can provide larger specific capacitances and higher energy densities [18–24].

Transition metal sulfides, especially bimetallic Ni–Co sulfides, have recently attracted considerable scientific and technological interest due to their excellent electrochemical performance [25–29]. On the one hand, these materials possess higher electrical conductivity, due to their lower band gap than that of ternary Ni–Co oxides [30–32]. On the other hand, the Ni–Co sulfides show richer Faradaic redox reactions than monometallic sulfides [33, 34], due to the contribution from the bimetallic elements, which results in higher specific capacitances [35–38]. However, similar to most other pseudocapacitive materials, the intrinsically low electronic conductivity, irreversible redox reactions, and structural instability of Ni–Co sulfides often limit their rate capability and cycle life after repeated Faradaic redox reactions [39, 40]. Although various bimetallic Ni–Co sulfides with different morphologies and components have been explored, their electronic conductivity and structural stability during long-term cycling are still unsatisfactory. To overcome these drawbacks, various carbonaceous materials were considered as hosts to load active materials and prepare composites. Several advantageous features have been identified for these carbon–NCS composites, including a highly open structure based on their carbon framework, an improved electrical conductivity of the electrode, an enhanced utilization of the active material, and a reduced dissolution of active materials into electrolytes. Compared to the widely used carbon nanotubes [41–44] and graphene [45–48], mesocarbon microbead (MCMB) materials are regarded as promising carbon hosts due to their low cost and easy synthesis, which are favorable for large-scale applications. The high conductivity of MCMB is expected to enhance the overall electrical conductivity of the composite, thus resulting in an improved electron transfer during electrochemical reactions. The spherical and open structure of MCMB can also provide a large number of adsorption sites to anchor other materials, which would enable a high loading of active materials. In addition, the robust solid structure of MCMB can produce a highly stable composite without massive agglomeration during electrochemical reactions, leading to a high structural stability of the electrode.

In this work, we adopted a facile two-step hydrothermal method to prepare a novel NiCo_2_S_4_@MCMB composite with urchin-like core–shell structure, by directly growing NiCo_2_S_4_ (NCS) nanoneedles on MCMBs. When evaluated as an electrode material for supercapacitors, the as-prepared NCS@MCMB composite displayed satisfactory electrochemical performance, which was attributed to its stable and open urchin-like structure. Moreover, the NCS@MCMB composite was also tested as an electrode material of asymmetric supercapacitors (ASCs), further confirming its potential in practical applications such as energy storage devices.

## Experimental Section

### Material Preparation

#### Synthesis of NCS@MCMB Precursors

The MCMB reactant was an industrial product prepared from coal tar pitch. The composites were synthesized by a facile two-step hydrothermal process: 101.6 mg of MCMB was first dispersed in 40 mL of deionized water by ultrasonication for 30 min, to obtain a suspension. Then, 1 mmol of Ni(NO_3_)_2_·6H_2_O, 2 mmol of Co(NO_3_)_2_·6H_2_O, and 12 mmol of urea were dissolved into the well-dispersed MCMB suspension, and the mixture was stirred for 20 min to obtain a clear dark pink solution. The suspension was placed into a 50-mL Teflon-lined stainless steel autoclave and maintained at 120 °C for 8 h. After cooling down to room temperature, the precipitate was collected and washed several times with deionized water and ethanol, followed by drying at 60 °C for 24 h in vacuum prior to the subsequent hydrothermal process.

#### Synthesis of NCS@MCMB

Na_2_S·9H_2_O (1.17 g) was dissolved in 40 mL of deionized water, followed by the addition of the obtained NCS@MCMB precursor. The suspension was stirred for 20 min, transferred into a 50 mL Teflon-lined stainless steel autoclave, and maintained at 160 °C for 12 h. After cooling down to room temperature, the precipitate was collected and washed several times with deionized water and ethanol, and then dried at 60 °C for 24 h in vacuum to yield a sample containing 75% NCS, labeled NCS@MCMB-75%.

For comparison, two additional samples, labeled NCS@MCMB-65% and NCS@MCMB-85%, were synthesized under the same reaction conditions but with NCS percentages of 65% and 85%, respectively. Pure NCS, CoS@MCMB, and NiS@MCMB were also synthesized under the same conditions.

### Material Characterization

The crystalline structure of the samples was identified by X-ray diffraction (XRD) using a Rigaku D/max 2500 V diffractometer (Rigaku, Japan) with Cu K_α_ radiation (*λ* = 1.5418 Å), operating voltage and current of 40 kV and 40 mA, respectively, and 2*θ* range from 20° to 70°. The morphology was characterized by Sirion 200 scanning electron microscopy (SEM, FEI, USA) and JEM 2100 F transmission electron microscopy (TEM, JEOL, Japan) with operating voltage 200 kV. X-ray photoelectron spectroscopy (XPS) measurements were taken using a Kratos Axis spectrometer (Kratos, Britain) with monochromatic Al K_α_ radiation (1486.71 eV), operating voltage and current of 15 kV and 10 mA, respectively, and a hemispherical electron energy analyzer. Raman spectra were recorder on an inVia spectrometer (Renishaw, Britain) with a 514 nm excitation source. Nitrogen adsorption/desorption isotherms at 77 K were measured using a Micromeritics Autosorb-iQ2-C instrument (Quantachrome, USA); specific surface areas were estimated by the Brunauer–Emmett–Teller (BET) method. Thermogravimetric analysis (TGA) was performed using Pyris 1 analyzer (PerkinElmer, USA) by heating under air flow from 30 to 900 °C at a rate of 10 °C min^−1^.

### Electrochemical Measurements

Electrochemical tests were performed on an electrochemical workstation (CHI 760e, Shanghai Chenhua, China), using a 3 M KOH aqueous solution at room temperature. The tests were performed in a three-electrode configuration with Pt foil, Ag/AgCl, and sample-modified nickel foam as counter, reference, and working electrodes, respectively. To prepare the working electrodes, the samples, acetylene black, and polyvinylidene fluoride (PVDF) were mixed in a 80:10:10 weight ratio in *N*-methyl-2-pyrrolidone (NMP) to form a slurry, which was pasted uniformly onto Ni foam (1 cm × 4 cm) with a thickness of 2 mm and dried in a vacuum oven at 120 °C for 12 h to remove the solvent. The mass loading of each electrode was about 2.5 mg (pasting surface area: 1 cm × 1 cm), corresponding to an areal mass loading capacity of 2.5 mg cm^−2^. The specific capacitance (F g^−1^) and current rate (A g^−1^) values were calculated based on the total mass of active material. Cyclic voltammetry (CV) measurements were taken from 0 to 0.5 V, at scanning rates from 2 to 100 mV s^−1^. Galvanostatic charge–discharge (GCD) measurements were taken from 0 to 0.5 V at current densities of 1–10 A g^−1^. Electrochemical impedance spectroscopy (EIS) measurements were taken by applying an alternate current voltage amplitude of 5 mV in the frequency range from 100 kHz to 0.01 Hz. All electrochemical measurements were taken at room temperature, and the specific capacitance (*C*) was calculated as *C* = (*I* × Δ*t*)/(Δ*V* × *m*), where *m* is the mass (g) of active material, *I* is the constant discharge current (A), Δ*V* is the applied potential window (V), and *t* denotes the discharge time (s).

The ASC device was fabricated using active carbon (AC) and the NCS@MCMB composite as the negative and positive electrodes, respectively. The device was fabricated using a CR2016-type coin cell with 3 M KOH solution as the electrolyte and one piece of cellulose paper as the separator. The energy density (*E*, Wh kg^−1^) and power density (*P*, W kg^−1^) of the NCS@MCMB//AC ASC device were calculated from the equations: *E* = *CV*^2^/2 and *P* = *E*/Δ*t*, respectively, where *V* is the applied potential and Δ*t* is the discharge time.

## Results and Discussion

**Fig. 1:**
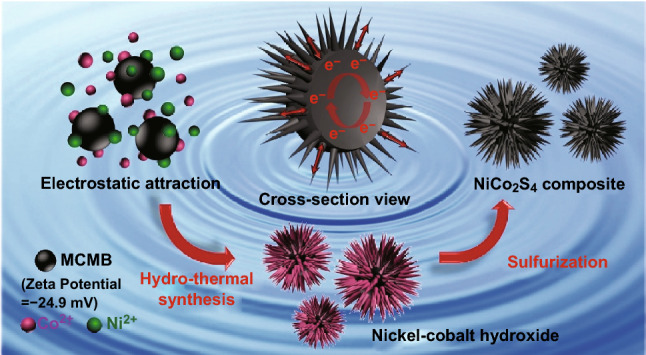
Schematic illustration of the synthesis procedure of the urchin-like NCS@MCMB composites

The mechanism of the two-step hydrothermal synthesis of the urchin-like NCS@MCMB composite is schematically illustrated in Fig. 1. First, MCMB was dispersed into a solution containing Ni^2+^ and Co^2+^. Owing to the negative zeta potential (i.e., − 24.9 mV) of MCMB in the solvent (Fig. S1), the metal Ni^2+^ and Co^2+^ cations were electrostatically attracted to the surface of the electronegative MCMB, which promoted the homogeneous nucleation and tight anchoring of the NCS precursors on MCMB in solution. During the first hydrothermal reaction, nickel–cobalt hydroxides (NiCo_2_(OH)_6_) crystallized in situ into nanoneedles located on the surface of MCMB, forming a core–shell urchin-like structure. The structure of the NCS@MCMB precursor was well preserved in the subsequent sulfurization during the second hydrothermal reaction, which allowed the transformation of hydroxides into sulfides [49], forming the stable urchin-like NCS@MCMB composite with core–shell structure. The open urchin-like structure enabled each NCS nanoneedle to effectively unfold, facilitating ion transfer with the electrolyte. At the same time, the MCMB served as a highly conductive electron collector, providing numerous electron transfer pathways. Therefore, the NCS@MCMB composite showed enhanced charge transfer and redox reaction activity, resulting in an increased specific capacitance and high-rate performance of the NCS@MCMB electrode.

**Fig. 2:**
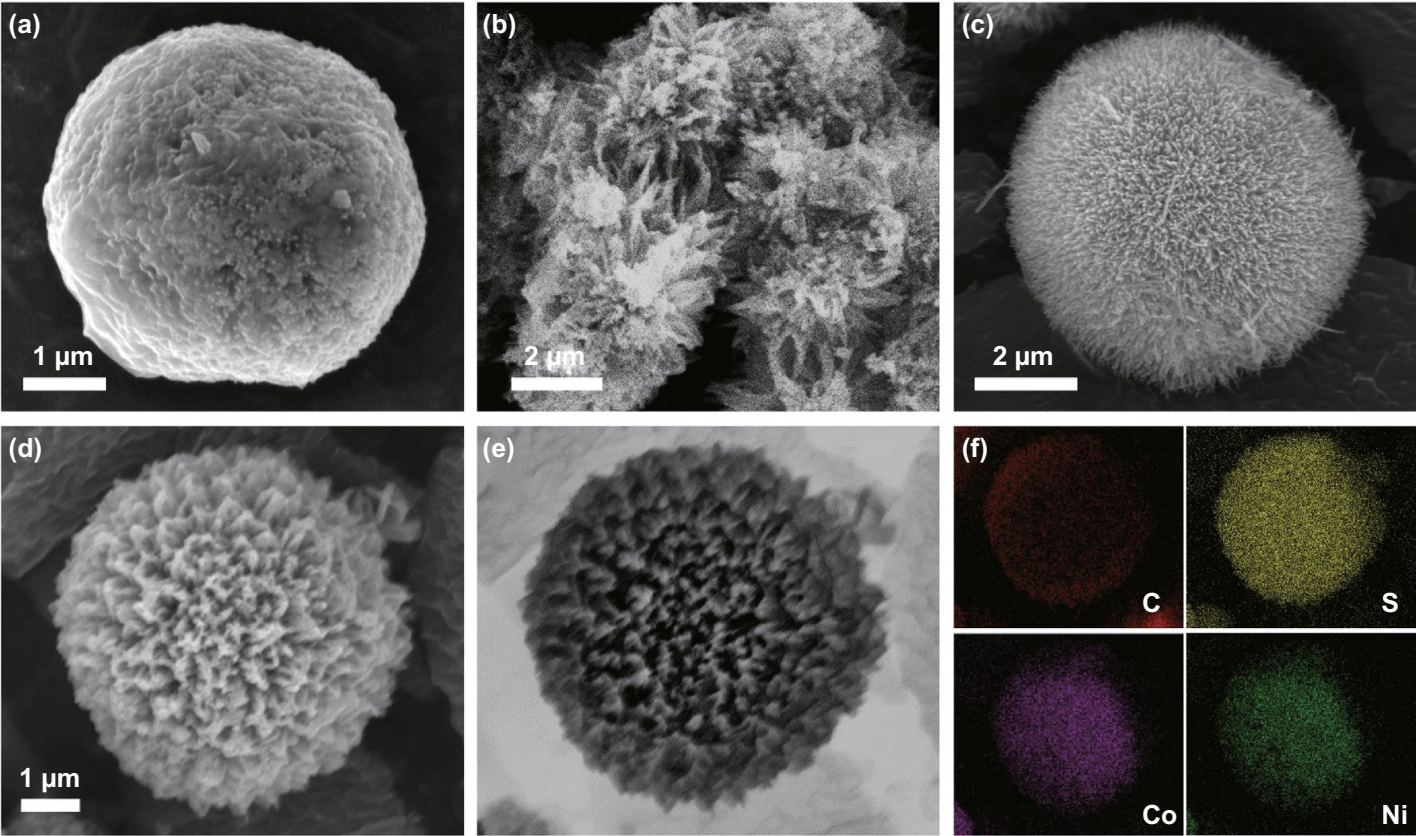
SEM images of **a** pure MCMB after calcination, **b** pure NCS, **c** NCS@MCMB precursor, and **d** NCS@MCMB composite. **e** SEM backscattered image of an individual NCS@MCMB sphere and **f** corresponding EDX elemental maps of C, S, Co, and Ni

The morphology changes occurring during the synthesis process were examined by SEM. Pure MCMB had an ideal spherical morphology, with a rough surface providing abundant adsorption sites (Fig. 2a). Pure NCS synthesized without the support of MCMB appeared as randomly arranged nanoneedles (Fig. 2b). When the NCS nanoneedles were synthesized in the presence of MCMB, their crystallization tended to occur on the MCMB surface, which contributed to the urchin-like arrangement of the nanoneedles. This is confirmed by Fig. 2c, which shows nickel–cobalt hydroxide nanoneedles uniformly anchored on MCMB and forming an urchin-like core–shell structure. The structure of the precursor was well retained after the subsequent sulfurization process, leading to the final urchin-like core–shell structure of NCS@MCMB (Fig. 2d), with a slightly larger size than that of pure MCMB. The energy-dispersive X-ray (EDX) spectrum of a single NCS@MCMB spherical particle in Fig. 2e, f shows that the C, S, Co, and Ni elements were uniformly distributed within the composite, further confirming its composition. Figure S2 displays a SEM image of the cross section of the electrode, revealing that the composite was closely attached to the Ni foam and retained its morphology; the average coating thickness was around 15 μm.

**Fig. 3:**
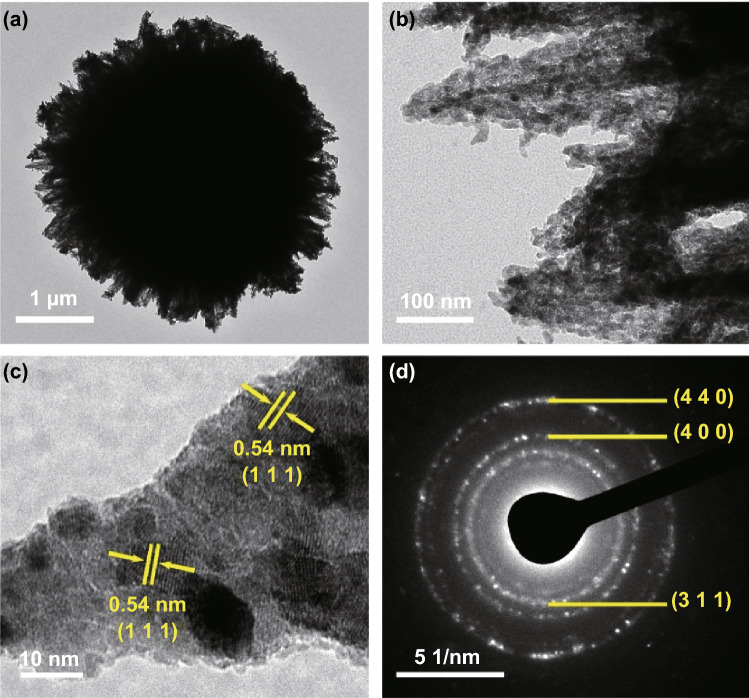
TEM images of **a** an individual NCS@MCMB sphere and **b** NCS nanoneedles anchored on it. **c** HRTEM image of NCS nanoneedles and **d** corresponding SAED patterns

The detailed morphology of the as-synthesized NCS@MCMB composite was further investigated by TEM and high-resolution TEM (HRTEM). The TEM image in Fig. 3a shows that numerous nanoneedles were distributed around the MCMB spheres, consistent with the SEM results. Furthermore, the high-magnification image in Fig. 3b highlights the porous structure of the NCS nanoneedles, which consisted of interconnected ultra-small NCS grains, formed from the anion exchange reaction during the sulfurization process [50]. The HRTEM image in Fig. 3c clearly shows a lattice fringe spacing of 0.54 nm, corresponding to the (111) plane of the spinel-structured NiCo_2_S_4_ phase. The polycrystalline nature of these NCS nanocrystals was also confirmed by the selected area electron diffraction (SAED) pattern displayed in Fig. 3d. Both SEM and TEM images highlight the open porous structure [50] of the composite, with numerous NCS nanoneedles tightly anchored on the MCMB surface, which is expected to result in a large specific surface area, abundant active sites for redox reactions, and superior electrochemical performances.

**Fig. 4:**
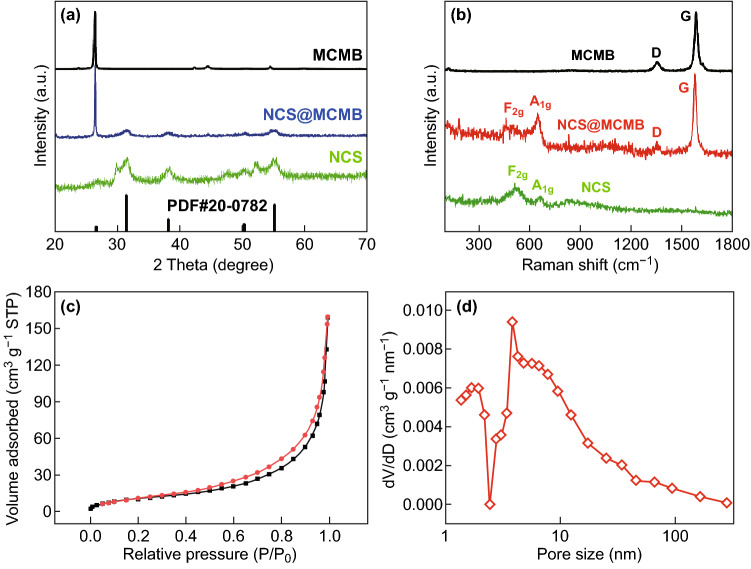
**a** XRD patterns and **b** Raman spectra of pure MCMB, NCS@MCMB, and pure NCS. **c** Nitrogen adsorption/desorption isotherm and **d** pore size distribution of NCS@MCMB composite

The XRD patterns of the NCS@MCMB composite and of the individual component materials are shown in Fig. 4a. The main diffraction peaks at 26.8°, 31.6°, 38.3°, 50.5°, and 55.3° in both NCS@MCMB and NCS alone could be, respectively, indexed to the (220), (311), (400), (511), and (440) planes of the standard spinel-structured NiCo_2_S_4_ (PDF #20-0782), indicating their well-crystallized structure. The EDX analysis (Fig. S3) indicated an Ni:Co:S atomic ratio of approximately 1:2:4 (14.10:29.28:56.62), consistent with the stoichiometric ratio of NiCo_2_S_4_. In addition, the same sharp diffraction peak at 26.5° was found for both pure MCMB and the composite, indicating the successful combination of NCS and MCMB. The structural features of the NCS@MCMB composite were further examined through the Raman spectra as shown in Fig. 4b. Two prominent peaks located at 1350 cm^−1^ (D band) and 1583 cm^−1^ (G band) were observed for both pure MCMB and NCS@MCMB. The D and G bands correspond to the disorder-induced features associated with *sp*^2^-bonded carbon and to the in-plane bond-stretching vibrations of *sp*^2^-hybridized carbon atoms, respectively [51, 52]. The intensity of D band was relatively low, implying a low number of defects in MCMB and a corresponding high conductivity. Furthermore, no obvious shift was observed in the G and D bands of pure MCMB and NCS@MCMB, which highlighted the stability of the MCMB structure after compositing with NCS. In addition, the peaks at 519 and 662 cm^−1^ in NCS and NCS@MCMB corresponded to the *F*_2g_ and *A*_1g_ modes of NiCo_2_S_4_, respectively [53, 54]. The N_2_ adsorption–desorption isotherm of the NCS@MCMB composite (Fig. 4c) shows a typical type IV isotherm with a distinct hysteresis loop in the relative pressure range of 0.4–1.0, as well as a decreasing slope at low relative pressures [55]. The BET specific surface area was calculated to be 69.8 m^2^ g^−1^. Furthermore, the NCS@MCMB composite showed typical pore sizes (Fig. 4d) in the ranges of 1.35–1.93 nm (indicating a small amount of micropores) and 3.8–9.5 nm (corresponding to a large number of mesopores), consistent with the porous structure of the NCS nanoneedles observed in the TEM images. The N_2_ adsorption–desorption isotherms and pore size distribution of pure NCS and MCMB were also tested and are shown in Fig. S4, which reveals much smaller specific surface areas and higher average pore sizes compared to those of the NCS@MCMB composite. The improved surface area of NCS@MCMB further confirmed the effective unfolding of NCS nanoneedles attached to MCMB, which resulted in an open and porous structure of the composite. Based on the TGA curves in Fig. S5, the full combustion of MCMB and the thermal decomposition of NCS occurred at 900 °C. The weight losses of pure NCS and NCS@MCMB were measured to be about 23% and 39%, respectively. Therefore, the content of MCMB in the NCS@MCMB-75% sample was calculated to be 21%, essentially in agreement with the expected content.

**Fig. 5:**
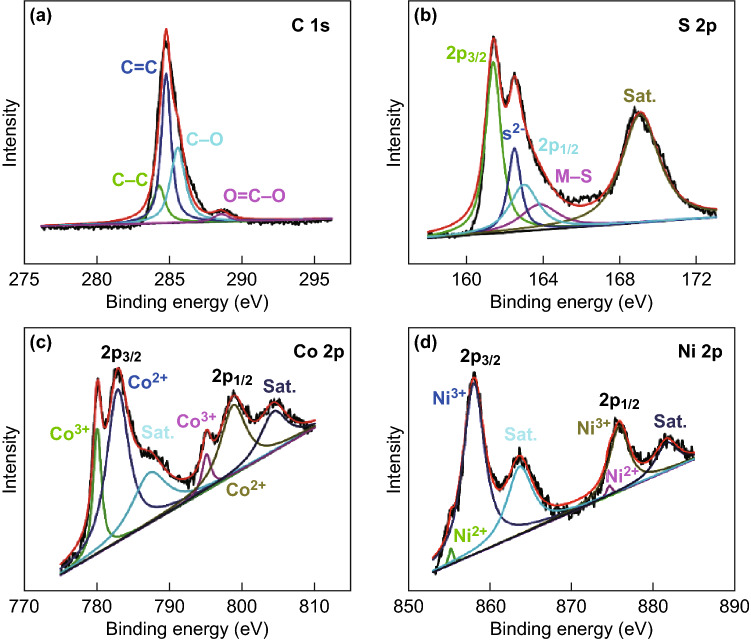
High-resolution XPS spectra of **a** C 1*s*, **b** S 2*p*, **c** Co 2*p*, and **d** Ni 2*p* levels

Detailed insight into the surface electronic states of the NCS@MCMB composite was obtained by XPS measurements; the spectra of all main elements, including C, S, Co, and Ni, are shown in Fig. 5. The C 1*s* spectrum (Fig. 5a) showed four major peaks corresponding to C–C, C = C, C–O, and O = C–O, indicating the presence of abundant oxygen-containing functional groups on the surface of MCMB. The existence of these functional groups played an important role in increasing the wettability of MCMB in the electrolyte and in improving the utilization of the specific surface area of MCMB, which further enhanced the pseudocapacitive performance and the specific capacitance of the electrode materials. The S 2*p* spectrum (Fig. 5b) showed three peaks at 169.1, 161.4, and 163.0 eV, corresponding to a shake-up satellite as well as to S 2*p*_3/2_ and S 2*p*_1/2_ states, respectively. The peak at 162.5 eV was attributed to low-coordinated divalent sulfide ions (S^2−^) on the surface, while the peak at 163.8 eV corresponded to typical metal–sulfur (M–S) bonds (Ni–S and Co–S) [29, 56]. The Co 2*p* spectrum (Fig. 5c) showed major peaks located at 782.8/798.8 eV for Co^2+^ and 780.0/795.0 eV for Co^3+^, with two shake-up satellites at 787.2 and 804.3 eV. The weak satellite peaks suggest that Co^3+^ was the dominant Co state. The Ni 2*p* spectrum (Fig. 5d) showed major peaks located at 855.2/874.6 eV for Ni^2+^ and 858.0/875.8 eV for Ni^3+^, with two shake-up satellites at 863.6 and 881.6 eV. The intense satellite peaks indicated that most Ni atoms were in the Ni^2+^ state. The coexistence of cations with different electronic states (Co^3+^/Co^2+^ and Ni^3+^/Ni^2+^) was accompanied by the presence of abundant active sites, which is beneficial for energy storage. The XPS results further confirmed the successful preparation of the NCS@MCMB composite.

**Fig. 6:**
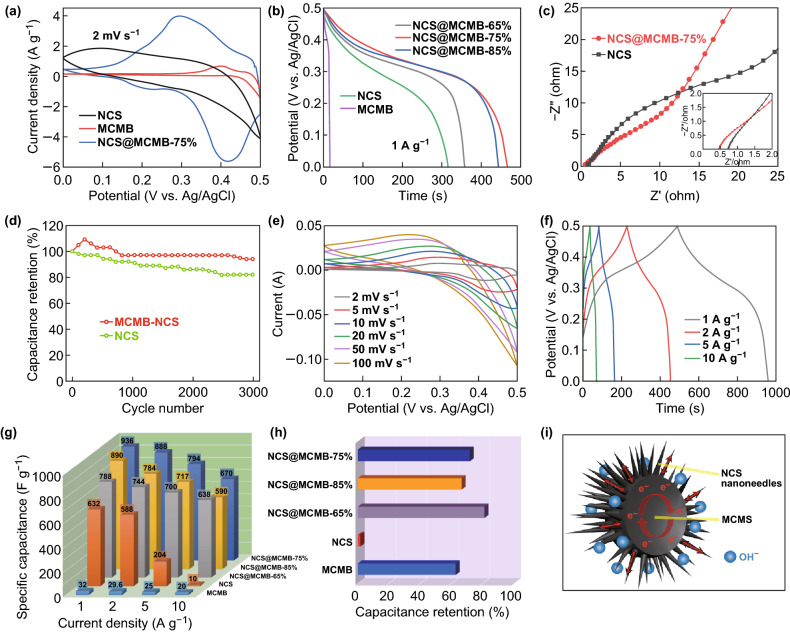
**a** CV curves at a scanning rate of 2 mV s^−1^, **b** GCD curves at a current density of 1 A g^−1^, **c** EIS spectra, and **d** cycling tests of NCS@MCMB and NCS. Typical **e** CV and **f** GCD curves of NCS@MCMB-75%. **g** Specific capacitance and **h** capacitance retention of NCS@MCMB, MCMB, and NCS at increased current densities. **i** Schematic diagrams illustrating the kinetic advantages of the NCS@MCMB composite in electrochemical energy storage

The electrochemical performances of NCS@MCMB composites with different loading amounts of NCS were evaluated by applying them as electrodes for supercapacitors. Pure NCS and MCMB electrodes were also tested for comparison. Figure 6a shows the CV curves of NCS@MCMB-75%, pure NCS, and MCMB electrodes recorded at the scan rate of 2 mV s^−1^. Among the three electrodes, the MCMB@NCS-75% one exhibited the strongest redox peaks, indicating the occurrence of Faradaic redox processes mainly related to the Co^2+^/Co^3+^ and Ni^2+^/Ni^3+^ redox couples [57]. Compared with those of pure NCS, the NCS@MCMB-75% composite presented a higher redox peak current and a larger area under the CV curves, suggesting that MCMB contributes to enhance the electrochemical activity and pseudocapacitive performances. The area under the CV curve suggests that the capacitance contribution from MCMB was negligible. Figure 6b shows the GCD curves of pure NCS, MCMB, and NCS@MCMB electrodes within the potential window of 0–0.5 V at 1 A g^−1^. Compared with those of the individual component materials, the longer discharge time in the composites indicates higher specific capacitances. This was attributed to the increased utilization rate of active materials due to the unique urchin-like structure of NCS@MCMB. Moreover, the longest discharge time of the NCS@MCMB-75% electrode reveals that an optimal NCS amount was loaded in this composite, maximizing the positive effects of the highly conductive MCMB to compensate the low capacitance. The resistance characteristics of the NCS@MCMB composite and NCS were determined by examining the Nyquist plots (Fig. 6c) obtained from the EIS measurements. Compared with pure NCS, the composite exhibited a larger slope in the low-frequency region, along with a smaller semicircle diameter and a lower real axis intercept at high frequencies, revealing lower diffusion resistance (*R*_w_), interfacial charge transfer resistance (*R*_ct_), and equivalent series resistance (*R*_s_) values, respectively [37, 58]. Overall, the EIS results reveal the improved electrical conductivity and enhanced Faradaic redox reactions of the NCS@MCMB composite, which are favorable for achieving the excellent rate capability and sustained cycling stability of the electrode.

The long-term cycling performance of the NCS and NCS@MCMB-75% electrodes were investigated at 5 A g^−1^ for 3000 cycles, and the corresponding results are shown in Fig. 6d. The capacitance retention of NCS@MCMB over 3000 cycles was 94%, larger than the 82% value obtained for pure NCS, which highlights an obvious improvement in cycling stability. The cycling stability of NCS@MCMB was also improved with respect to that of previously reported NCS electrodes [59–62], demonstrating its good structural stability during electrochemical reactions. Apart from the high electrical conductivity revealed by the EIS results, the sustained cycling stability of the NCS@MCMB composite also benefitted from the tight integration, better interfacial interaction, and improved electrical contact between NCS nanoneedles and MCMB. In addition, MCMB served as an ideal substrate material to reduce the agglomeration of NCS nanoneedles during the long-term cycling tests, resulting in a highly stable composite structure.

As shown in Fig. 6e, the CV curves of the NCS@MCMB-75% electrode were further evaluated at different scan rates ranging from 2 to 100 mV s^−1^, within the potential window of 0–0.5 V. The 50-fold increase in scan rate resulted in the anode peak shifting slightly from 0.29 to 0.23 V, demonstrating the excellent high-rate reversibility of the composite electrode [63, 64]. The obvious redox peaks in the CV curves confirmed the pseudocapacitive behavior of NCS, which was responsible for its large capacitance [62, 65]. Similar Faradaic redox peaks were observed in both NCS@MCMB-65% and NCS@MCMB-85% composites (Fig. S6b, d), which also exhibited more obvious redox peaks compared with those of pure NCS or MCMB (Fig. S7b, d), further confirming the enhanced pseudocapacitive characteristics of NCS@MCMB. The nonlinear trend of the GCD curves at high current densities ranging from 1 to 10 A g^−1^ (Fig. 6f) further confirmed the Faradaic characteristic of NCS@MCMB. The GCD curves presented voltage plateaus at around 0.35 V consistent with the CV curves, implying the high reversibility of the composite. The specific capacitance values at different current densities and the capacitance retention values (Fig. 6g, h) were calculated from the GCD measurements of various electrodes (Figs. 6f, S6a, c, and S7a, c). Although pure NCS possessed a high capacitance of 632 F g^−1^ at 1 A g^−1^, this value sharply decreased with the gradual increase in current density (1.6% capacitance retention at 10 A g^−1^). In contrast, MCMB exhibited a lower specific capacitance (32 F g^−1^ at 1 A g^−1^) but also an excellent rate performance (62.5% capacitance retention). Therefore, the NCS@MCMB composite exhibited a synergistic effect between the two components, which led to greatly improved capacitance and rate performance. Among the composites with different NCS contents, the NCS@MCMB-75% electrode displayed the highest specific capacitances, with values of 936, 888, 794, and 670 F g^−1^ at current densities of 1, 2, 5, and 10 A g^−1^, respectively. This indicated that about 71.58% of the specific capacitance was retained after a tenfold increase in current density, indicating excellent rate capability. In contrast, the lower capacitance of NCS@MCMB-85% (890 F g^−1^ at 1 A g^−1^) might be due to the underutilization of the NCS grains that were not loaded on MCMB. Despite a slightly higher capacitance retention of 80.96% than that of NCS@MCMB-75%, the NCS@MCMB-65% composite displayed a much lower capacitance (788 F g^−1^ at 1 A g^−1^), due to its lower NCS content. Therefore, we inferred that the NCS@MCMB-75% composite possessed an optimal NCS:MCMB ratio, which achieved a favorable balance between high capacitance and high-rate performance. The superior electrochemical performances of the NCS@MCMB composites originated from their unique urchin-like structure (Fig. 6i). On the one hand, the NCS nanoneedles were sufficiently unfolded to gain full contact with the OH^−^ groups in the electrolyte, thereby promoting the Faradaic redox processes on the NCS nanoneedles. On the other hand, the highly conductive MCMB served as a substrate to support individual NCS nanoneedles, which greatly enhanced the electron transfer in the composite. The favorable charge transfer in the NCS@MCMB composite resulted in high specific capacitances, promising high-rate performance, and excellent long-term cycling stability. We also prepared monometallic CoS@MCMB and NiS@MCMB composites, and their CV and GCD curves are shown in Fig. S8. Both materials showed smaller capacitances than NCS@MCMB (391 F g^−1^ for CoS@MCMB and 474 F g^−1^ for NiS@MCMB), demonstrating the beneficial effect of bimetallic Ni–Co sulfides.

**Fig. 7:**
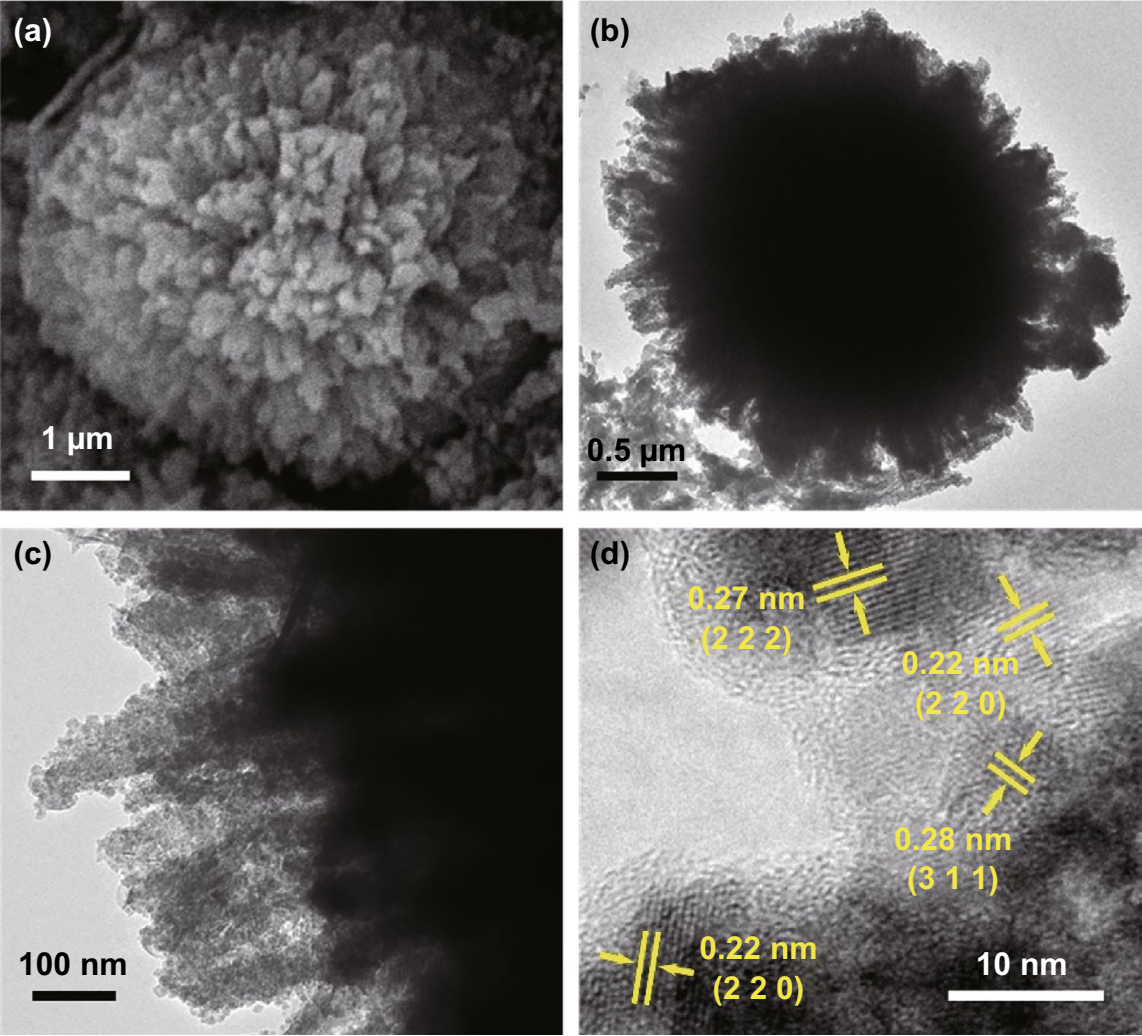
**a** SEM, **b**, **c** TEM, and **d** HRTEM images of NCS@MCMB after cycling

The stability of the composite structure could be further confirmed by inspecting the morphology after cycling. As shown in Fig. 7a, the SEM image highlights an unchanged urchin-like spherical shape without obvious collapse and aggregation, in good agreement with the TEM image of an individual NCS@MCMB sphere (Fig. 7b). Moreover, the enlarged image of a part of the urchin-like NCS@MCMB sphere (Fig. 7c) shows that each NCS nanoneedle retained their porous and perfect needlelike structure without breaking. These results illustrate the strong interaction between the NCS nanoneedles and MCMB, which allowed the composite to maintain its integrated urchin-like morphology after the long-term cycling process. Moreover, clear lattice fringe spacings were observed at 0.22, 0.27, and 0.28 nm in Fig. 7d, corresponding to the (220), (222), and (311) planes, respectively, indicating that the crystal structure and polycrystalline feature of the NCS nanoneedles did not change during the pseudocapacitive reactions. No obvious peak shift was found in the XPS C 1*s*, S 2*p*, Co 2*p*, and Ni 2*p* spectra after cycling (Fig. S9), further confirming the excellent compositional and structural stability of the composite. The high structural stability of the NCS@MCMB composite was responsible for the excellent cyclic reversibility and high capacitance retention of the electrode.

**Fig. 8:**
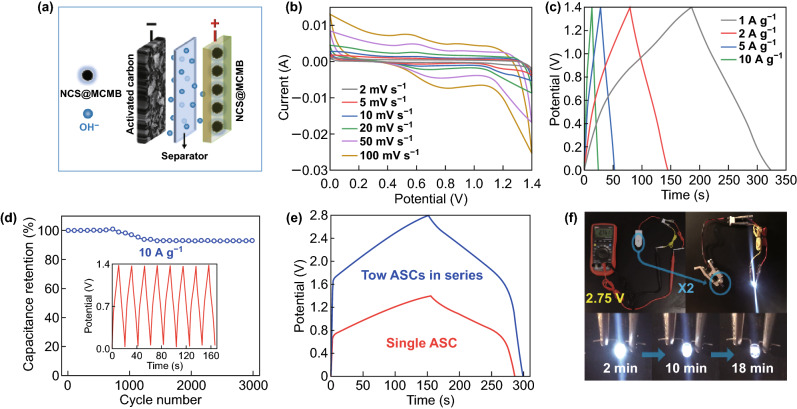
**a** Schematic illustration of the structure of the NCS@MCMB//AC ASC device. **b**, **c** Typical CV and GCD curves of the ASC device. **d** Cycling test of the ASC device at 10 A g^−1^ and GCD curves in the last eight cycles (inset of **d**). **e** GCD curves of single ASC and two ASCs connected in series. **f** Photographs of two ASC devices connected in series, powering a red LED indicator

To further evaluate the potential application of the NCS@MCMB composite, we assembled an asymmetric supercapacitor device with NCS@MCMB as the positive electrode and active carbon as the negative electrode (Fig. 8a). In order to ensure the charge storage balance between the positive and negative electrodes (*Q*_+_ = *Q*_−_), the masses of the active materials should follow the equation (*m*_−_/*m*_+_) = (*C*_+_ × Δ*V*_+_)/(*C*_−_ × Δ*V*_−_), where the “+” and “_−_” subscripts correspond to the positive and negative charge carriers, *m* is the mass of active material, *C* is the specific capacitance of the electrode, and Δ*V* is the potential range for the electrochemical reaction [66]. Based on the 105 F g^−1^ specific capacitance of AC calculated from the GCD curve in Fig. S10a, the optimal NCS@MCMB:AC mass ratio in the NCS@MCMB//AC ASC was calculated to be about 4.5. Figure S10b shows the CV curves of AC and NCS@MCMB electrodes, whose stable voltage windows were identified as − 0.9 to 0 V and 0 to 0.5 V, respectively, corresponding to the 0–1.4 V voltage window in the ASC device (Fig. 8b). As shown in Fig. 8b, the CV curve of the ASC device suggests the presence of both electric double-layer capacitance and pseudocapacitance. Similar shapes, without evident distortion, were retained by the CV curves as the scanning rate was increased, indicating excellent capacitive characteristics and rate capability. The GCD curves of the ASC device at current densities ranging from 1 to 10 A g^−1^ (Fig. 8c) showed quasi-triangular shapes with good symmetry, suggesting excellent reversibility and capacitive characteristics. According to the GCD curves, the specific capacitances of the ASC at current densities of 1, 2, 5, and 10 A g^−1^ were calculated to be 97.79, 93.14, 85.71, and 78.57 F g^−1^, respectively, confirming the excellent capacitive characteristics and rate capability. A reasonable total capacitance (*C*_T_, 97.79 F g^−1^) of the ASC device is estimated from Eq. 1/*C*_T_ = 1/*C*_p_ + 1/*C*_n_, where *C*_p_ and *C*_n_ are the capacitances of the positive (936 F g^−1^) and negative (105 F g^−1^) electrodes, respectively. Furthermore, the long-term stability was investigated at the current density of 10 A g^−1^ (Fig. 8d). The results reveal a superior cycling stability, with 96.2% capacitance retention after 3000 cycles. The GCD curves of the last eight cycles (shown in the inset of Fig. 8d) remained almost unchanged, which demonstrated an excellent cyclic reversibility. The Ragone plots (energy density vs. power density) shown in Fig. S11 represent important tools for evaluating the potential application of an ASC device. They show that the NCS@MCMB//AC ASC delivered an energy density of 26.62 Wh kg^−1^ at a low power density of 699.98 W kg^−1^ (1 A g^−1^) and an energy density of 21.39 Wh kg^−1^ at a high power density of 7000 W kg^−1^ (10 A g^−1^), which are among the best performance reported for ASCs to date. To further demonstrate the excellent performance of the NCS@MCMB composite, two ASC devices were connected in series to power a white light-emitting diode (LED, about 3.0 V). As shown in the GCD curves (Fig. 8e), the operating voltage of the two ASC devices could reach 2.8 V, consistent with the voltage monitored by a digital multimeter (Fig. 8f), thus achieving the voltage required to power the LED. In fact, as shown in Fig. 8f, after charging for 20 s, the LED was powered for as long as 18 min, further evidencing the excellent performance of the devices and the potential applications of the NCS@MCMB composite.

The outstanding electrochemical performances of the MCMB@NCS composite could be ascribed to its unique microstructure and the synergistic effect of MCMB and NCS. First, the bimetallic sulfides possessed abundant active sites for the Faradaic redox reactions, resulting in a high specific capacitance of the composite. Second, the MCMB component imparted excellent electrical conductivity to the composite, leading to rapid electron transfer and high-rate performance. Third, the composite presented a highly porous structure, which enhanced cationic diffusion in the electrolyte, thereby leading to improved ion transport efficiency. Fourth, the NCS nanoneedles were uniformly anchored on the MCMB surface, which allowed them to be fully unfolded and effectively contribute to the Faradaic redox reactions, thereby leading to a greatly increased utilization ratio of NCS and superior pseudocapacitive characteristics. Moreover, the stable composite structure with a strong NCS-MCMB interaction allowed maintaining a high structural stability during the redox reactions, thereby leading to highly reversible electrochemical reactions and significantly enhanced cycling performance.

## Conclusions

In summary, a simple two-step hydrothermal strategy was developed to synthesize a NCS@MCMB composite with unique urchin-like core–shell structure, in which bimetallic NCS nanoneedles were uniformly anchored on MCMB. When evaluated as an electrode material for supercapacitors, the unique structure and synergistic effects endowed the composite electrode with a high specific capacitance of 936 F g^−1^ at 1 A g^−1^, high cyclic stability (96.2% capacitance retention after 3000 cycles), and promising rate capability (670 F g^−1^ at 10 A g^−1^). An asymmetric supercapacitor was also fabricated, which showed great promise for practical applications in the energy storage field. Moreover, the present fabrication procedure and device architecture could be extended to other active bimetallic and carbonaceous materials and promote future applications in high-performance electrochemical energy storage/conversion devices.

## Electronic supplementary material

Below is the link to the electronic supplementary material.

Supplementary material 1 (PDF 1614 kb)
