# Supplementary material for: Bimetallic NiCo2S4 Nanoneedles Anchored on Mesocarbon Microbeads as Advanced Electrodes for Asymmetric Supercapacitors
Source: Nanomicro Lett. 2019 Apr 23;11:35. doi: 10.1007/s40820-019-0265-1 (PMC7770863; doi:10.1007/s40820-019-0265-1)
Supplement: Supplementary file 1 — Supplementary material 1 (PDF 1614 kb) [file 40820_2019_265_MOESM1_ESM.pdf]

Supporting Information for

Bimetallic $NiCo_2S_4$ Nanoneedles Anchored on Mesocarbon Microbeads as Advanced Electrodes for Asymmetric Supercapacitors

Yu Zhang[1], Yihe Zhang[1,]*, Yuanxing Zhang[1], Haochen Si[1], Li Sun[1,] *

[1]Beijing Key Laboratory of Materials Utilization of Nonmetallic Minerals and Solid Wastes, National Laboratory of Mineral Materials, School of Materials Science and Technology, China University of Geosciences, Beijing 100083, People's Republic of China

*Corresponding authors. E-mail: zyh@cugb.edu.cn (Yihe Zhang); sunli@cugb.edu.cn (Li Sun)

Supplementary Figures

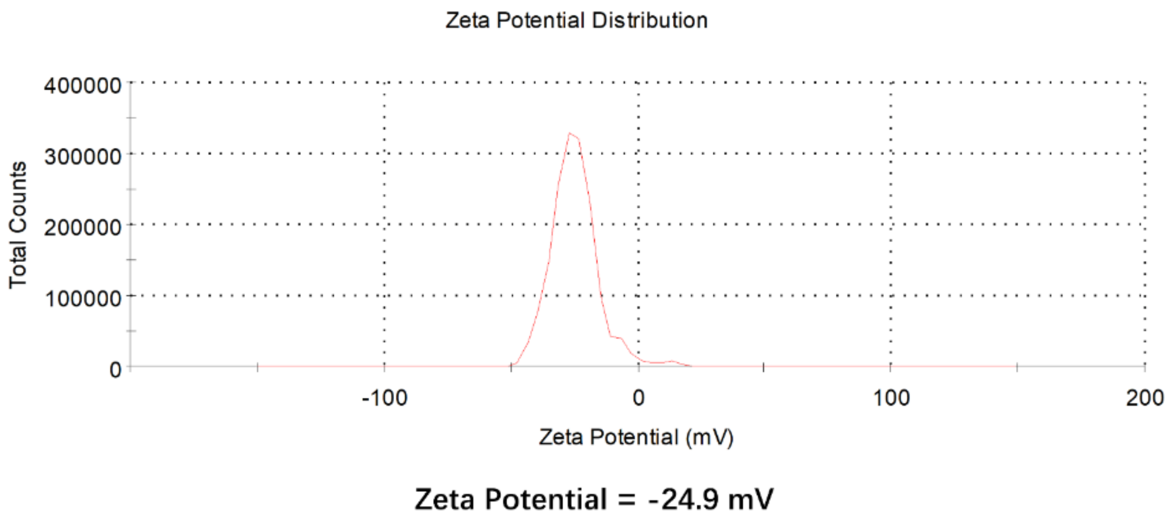

Fig. S1 Zeta potential of pure MCMB

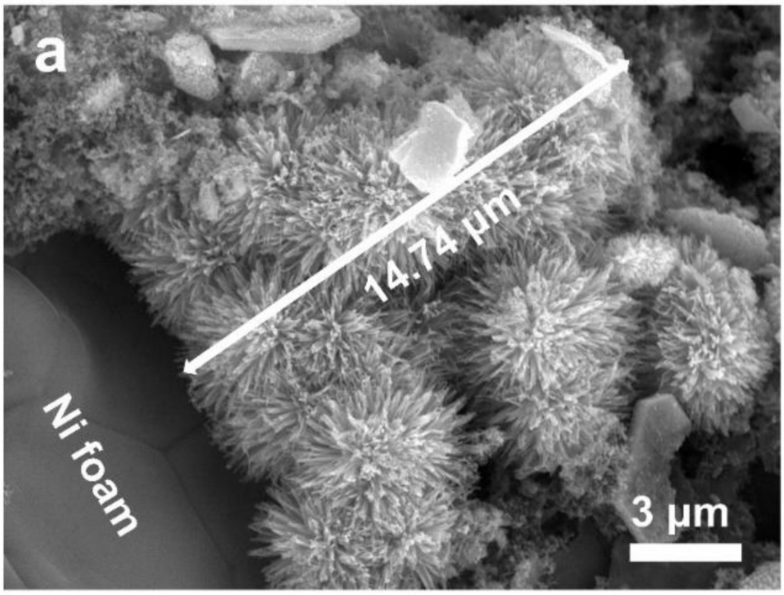

Fig. S2 Cross section of the NCS@MCMB electrode

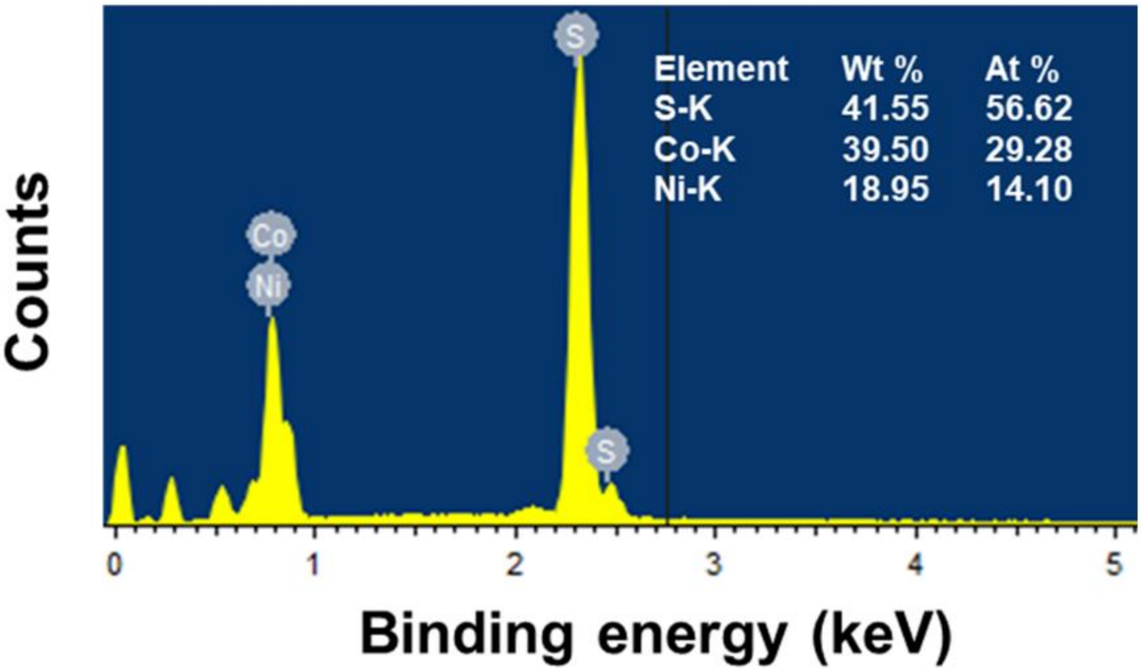

Fig. S3 EDX spectra of NCS@MCMB composite

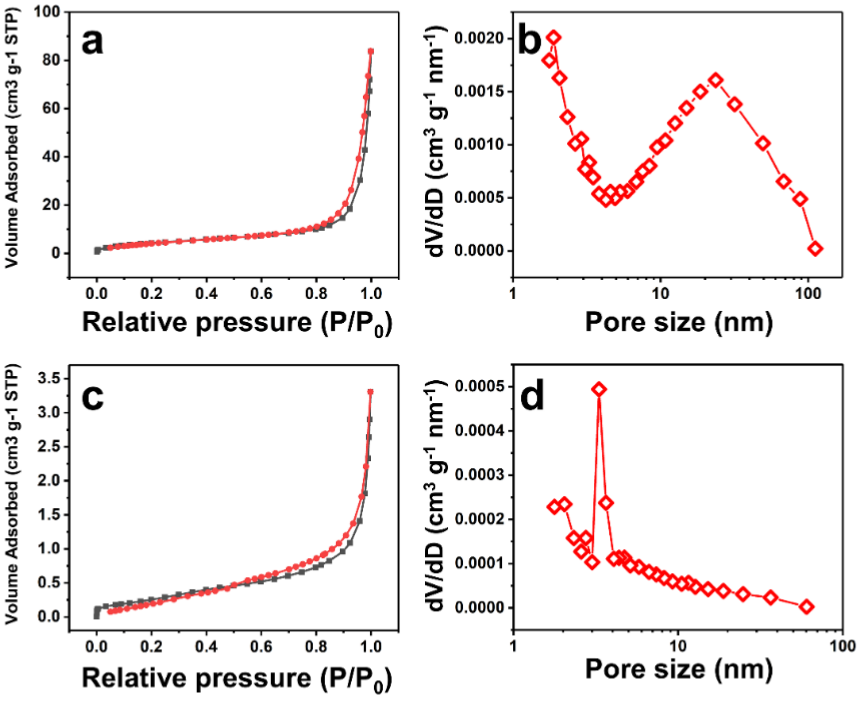

Fig. S4 a, c Nitrogen adsorption/desorption isotherms and **b, d** pore-size distribution of pure NCS and MCMB

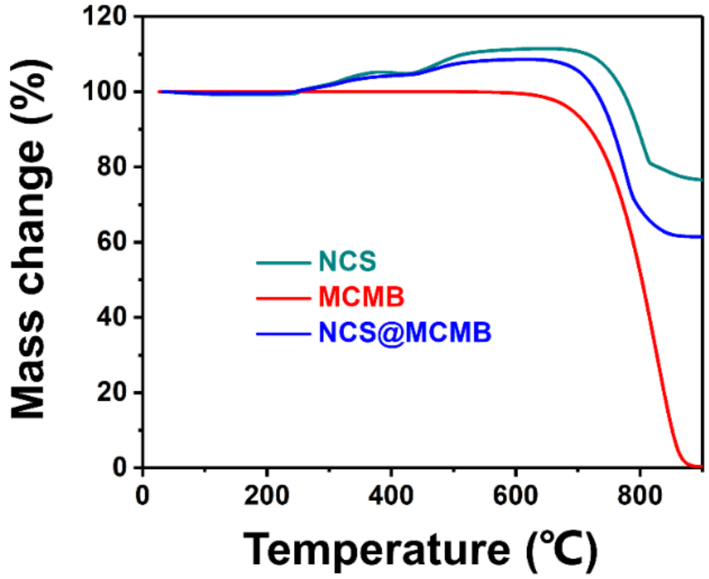

Fig. S5 TGA curves of pure NCS, MCMB, and NCS@MCMB-75%

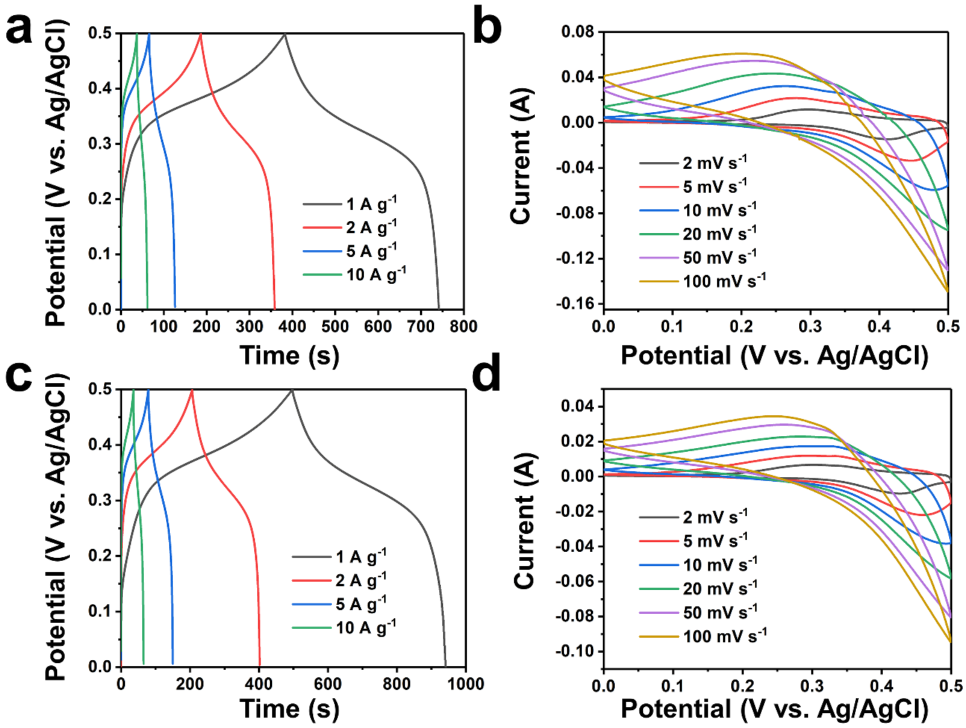

Fig. S6 a, c GCD curves and **b, d** CV curves of NCS@MCMB-65% and NCS@MCMB-85%

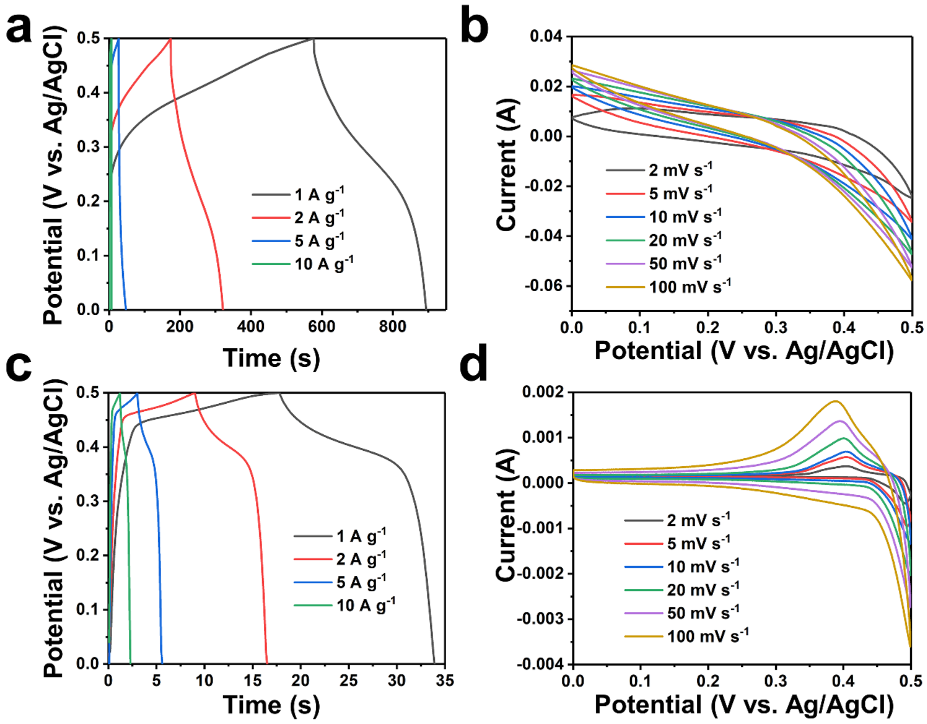

Fig. S7 a, c GCD curves and **b, d** CV curves of pure NCS and MCMB

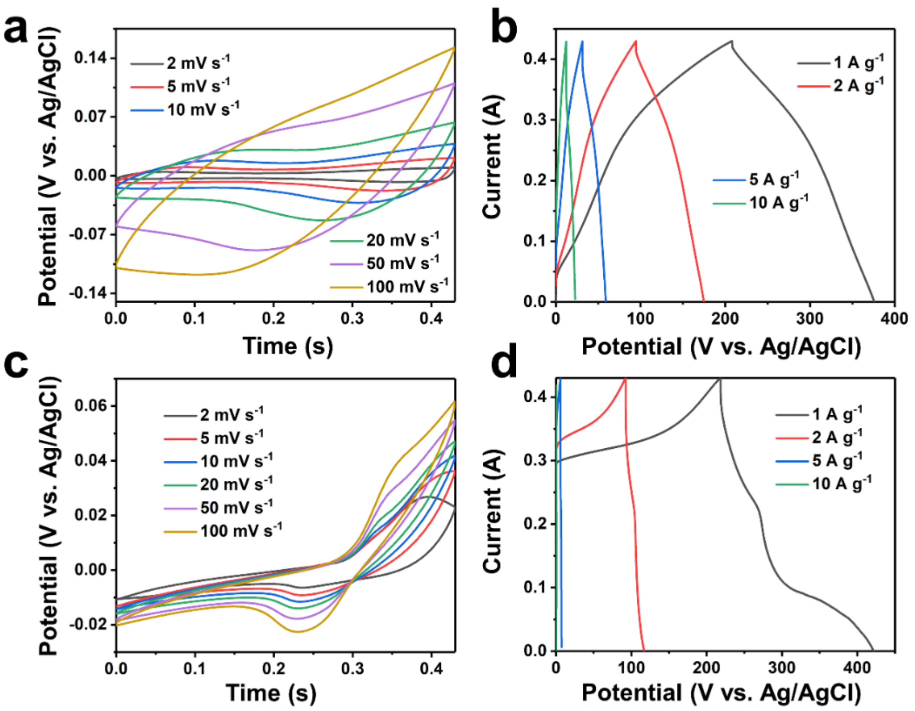

Fig. S8 a, c CV curves and **b, d** GCD curves of CoS@MCMB and NiS@MCMB

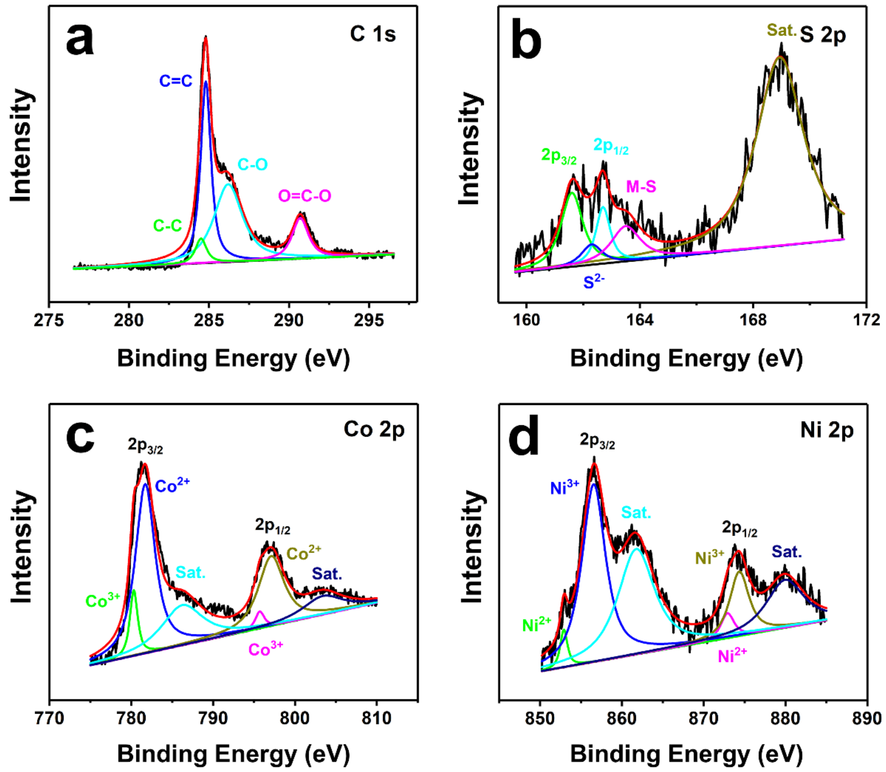

Fig. S9 XPS high-resolution spectra after cycling of **a** C 1s, **b** S 2p, **c** Co 2p, and **d** Ni 2p

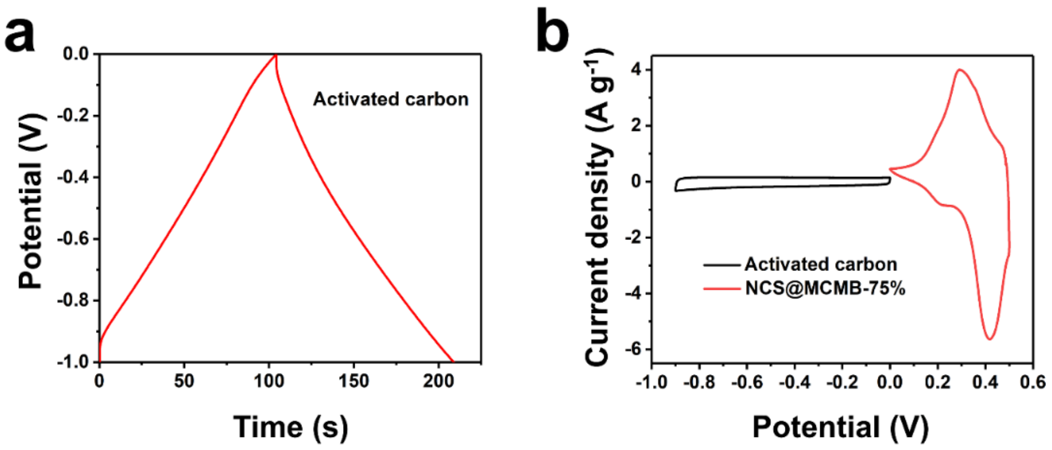

Fig. S10 a GCD curve of activated carbon at a current density of 1 A g^{-1}. **b** CV curves of activated carbon and NCS@MCMB-75% at a scan rate of 2 mV s^{-1}

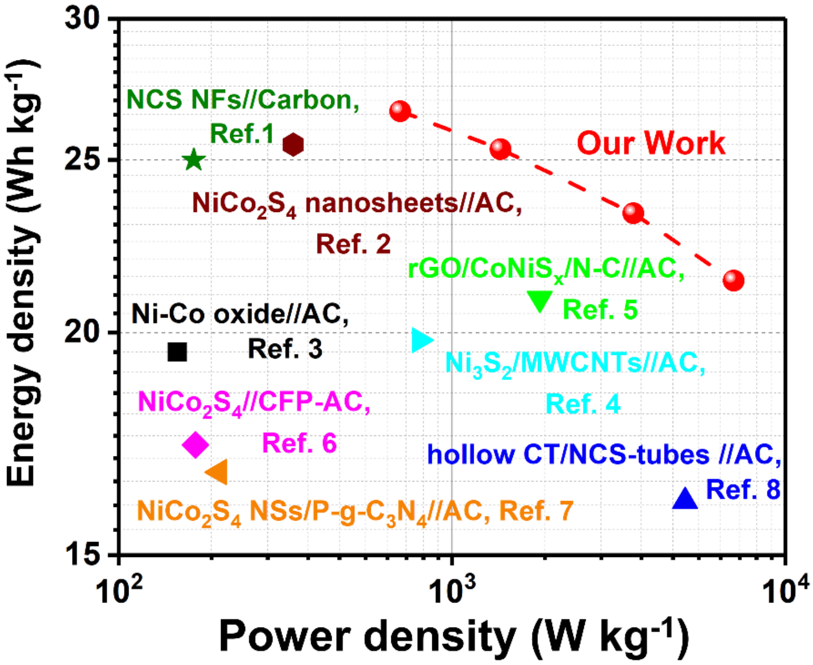

Fig. S11 Ragone plot of the NCS@MCMB//AC ASC

Supplementary References

[S1]S. Hussain, T.M. Liu, M.S. Javed, N. Aslam, N. Shaheen, S.Q. Zhao, W. Zeng, J.X. Wang, Amaryllis-like $NiCo_2S_4$ nanoflowers for high-performance flexible carbon-fiber-based solid-state supercapacitor. Ceram. Int. **42**(10), 11851-11857 (2016). https://doi.org/10.1016/j.ceramint.2016.04.107

[S2]Z.B. Wu, X.L. Pu, X.B. Ji, Y.R. Zhu, M.J. Jing, Q.Y. Chen, F.P. Jiao, High energy

density asymmetric supercapacitors from mesoporous $NiCo_2S_4$ nanosheets. Electrochim. Acta **174**, 238-245 (2015). https://doi.org/10.1016/j.electacta.2015.06.011

[S3]H.L. Wang, C.M.B. Holt, Z. Li, X.H. Tan, B.S. Amirkhiz, Z.W. Xu, B.C. Olsen, T. Stephenson, D. Mitlin, Graphene-nickel cobaltite nanocomposite asymmetrical supercapacitor with commercial level mass loading. Nano Res. **5**(9), 605-617 (2012). https://doi.org/10.1007/s12274-012-0246-x

[S4]C.S. Dai, P.Y. Chien, J.Y. Lin, S.W. Chou, W.K. Wu, P.H. Li, K.Y. Wu, T.W. Lin, Hierarchically structured Ni_3S_2/carbon nanotube composites as high performance cathode materials for asymmetric supercapacitors. ACS Appl. Mater. Interfaces **5**(22), 12168-12174 (2013). https://doi.org/10.1021/am404196s

[S5]Q.D. Chen, J.K. Miao, L. Quan, D.P. Cai, H.B. Zhan, Bimetallic $CoNiS_x$ nanocrystallites embedded in nitrogen-doped carbon anchored on reduced graphene oxide for high-performance supercapacitors. Nanoscale **10**(8), 4051-4060 (2018). https://doi.org/10.1039/c7nr08284c

[S6]X.H. Xiong, G. Waller, D. Ding, D.C. Chen, B. Rainwater, B.T. Zhao, Z.X. Wang, M.L. Liu, Controlled synthesis of $NiCo_2S_4$ nanostructured arrays on carbon fiber paper for high-performance pseudocapacitors. Nano Energy **16**, 71-80 (2015). https://doi.org/10.1016/j.nanoen.2015.06.018

[S7]Z.C. Li, L. Wu, L.B. Wang, A.J. Gu, Q.F. Zhou, Nickel cobalt sulfide nanosheets uniformly anchored on porous graphitic carbon nitride for supercapacitors with high cycling performance. Electrochim. Acta **231**, 617-625 (2017). https://doi.org/10.1016/j.electacta.2017.02.087

[S8]N.N. Wang, Y.J. Wang, S.Z. Cui, H.W. Hou, L.W. Mi, W.H. Chen, A hollow tube-on-tube architecture of carbon-tube-supported nickel cobalt sulfide nanotubes for advanced supercapacitors. ChemNanoMat **3**(4), 269-276 (2017). https://doi.org/10.1002/cnma.201700016
